# Associations between seven-year C-reactive protein trajectory or pack-years smoked with choroidal or retinal thicknesses in young adults

**DOI:** 10.1038/s41598-021-85626-3

**Published:** 2021-03-17

**Authors:** Samantha Sze-Yee Lee, Darren John Beales, Fred K. Chen, Seyhan Yazar, David Alonso-Caneiro, David A. Mackey

**Affiliations:** 1 Centre for Ophthalmology and Visual Science (Incorporating Lions Eye Institute), University of Western Australia, 2 Verdun St, Nedlands, WA 6009 Australia; 2 School of Physiotherapy and Exercise Science, Curtin University, Perth, WA Australia; 3 Department of Ophthalmology, Royal Perth Hospital, Perth, WA Australia; 4 Single Cell and Computational Genomics Lab, Garvan Institute of Medical Research, Darlinghurst, NSW Australia; 5 Contact Lens and Visual Optics Laboratory, Centre for Vision and Eye Research, School of Optometry and Vision Science, Queensland University of Technology (QUT), Brisbane, QLD Australia; 6 Centre for Eye Research Australia, Royal Victorian Eye and Ear Hospital, University of Melbourne, Melbourne, VIC Australia; 7 School of Medicine, Menzies Research Institute Tasmania, University of Tasmania, Tasmania, Australia

**Keywords:** Epidemiology, Inflammation, Eye diseases, Risk factors

## Abstract

Inflammation and cigarette smoking predispose to macular diseases, and choroidal and retinal thinning. We explored the choroidal and retinal thicknesses in young adults against their 7-year C-reactive protein (CRP) level trajectory and pack-years smoked. Participants from the Raine study, a longitudinal cohort study, had serum CRP levels analysed at the 14-, 17-, and 20-year follow-ups. Group-based trajectory modelling was used to classify participants according to their 7-year CRP levels. At the 20-year follow-up (at 18–22 years old), participants completed questionnaires on their smoking history, and underwent optical coherence tomography imaging to obtain their choroidal and retinal thicknesses at the macula. Three CRP trajectories were identified: consistently low CRP levels (78% of sample), increasing (11%), or consistently high (11%). 340 and 1035 participants were included in the choroidal and retinal thickness analyses, respectively. Compared to those in the “Low” trajectory group, participants in the “Increasing” and “High” groups had 14–21 μm thinner choroids at most macular regions. Every additional pack-year smoked was linked with a 0.06–0.10 μm thinner retina at the inner and outer macular rings, suggesting a dose-dependent relationship between smoking and thinner retinas. These associations may suggest that an increased risk of future visual impairment or eye disease associated with these risk factors may be present since young adulthood.

## Introduction

Inflammation is implicated in the pathogenesis of several ocular disease, including ocular surface disease, age-related macular degeneration (AMD), and uveitis^1,2^. Choroiditis or retinitis may also occur as a result of systemic inflammatory disorders^3,4^. Even in the absence of choroidal or retinal inflammation, changes in the thicknesses of these structures have been noted in systemic autoimmune conditions^4^. Studies have reported that the choroid tends to thicken during the active phase of a systemic autoimmune disease such as Behçet disease^5^ and systemic lupus erythematosus^3^. With chronic or recurrent inflammation, the choroid becomes thinner, which is believed to be due to fibrosis, prolonged ischemia, and atrophy^4^. Additionally, case–control studies^6,7^ have found thinner maculas, inner retinal layers, ganglion cell complexes, and nerve fibre layers in patients with systemic autoimmune disorders compared to age-matched healthy controls. However, there is limited information in the literature on how the retinal thickness may vary according to disease status (i.e. active or remission). Researchers believe that vasculitis associated with these conditions results in retinal microstructure infarctions, which subsequently lead to cell death and retinal thinning^6,7^.

Choroidal and retinal thickness changes have also been noted in AMD, the leading cause of visual impairment in developed countries^8^, as well as in other inflammation-related diseases of the macula. For example, individuals with AMD tend to have thinner retinas^9^ and choroids^10^ compared to those with healthy eyes. On the other hand, various forms of macular oedema intrinsically result in thickening of the central retina, while thicker choroids have been noted in polypoidal choroidal vasculopathy. Histological experiments have revealed accumulations of C-reactive protein (CRP), a highly sensitive systemic marker of inflammation and tissue damage^11^, in the cytoplasm of retinal pigmented epithelium cells with overlying drusen^12^, a sign of AMD. It has been proposed that repeated attack of the retinal pigmented epithelium by inflammatory cells leads to the development of drusen and AMD^13^. Indeed, a link between higher serum CRP levels and presence of AMD have been reported in cross-sectional studies^14,15^, although others have failed to find a significant relationship^16,17^. The longitudinal Rotterdam Study^18^ and the Women’s Health Study^19^ reported that higher serum CRP levels at baseline were associated with future diagnosis of AMD in older adults (mean follow-up durations of 8 and 10 years, respectively). Further, a meta-analysis estimated that elevated serum levels of CRP are associated with a 1.7-fold increase in AMD risk^20^.

Elevated levels of CRP have also been noted in cigarette smokers^21^, who are known to be at higher risk of AMD. A meta-analysis estimated that current smokers have a 1.8 to 3.6 times increased risk of AMD relative to non-smokers^22^. Studies have also observed thinner choroids^23^ and retinas^23^ in middle-aged smokers compared to age-matched non-smokers, although other reports failed to find significant difference in thicknesses between groups^24,25^.

Given that choroidal and retinal thicknesses may be altered in those with autoimmune diseases or macular disease, and the role of inflammation in these conditions, we should consider the possibility that a chronic sub-clinical inflammatory state (as measured by serum CRP levels) maybe associated with variations in choroidal or retinal thicknesses even in the absence of a clinical diagnosis of an ocular or a systemic inflammatory disorder. A common limitation of many previous studies^14–17^ analysing the association between CRP and AMD was that serum CRP was only measured at a single time-point, usually at the same time that the AMD was diagnosed, which does not account for earlier CRP levels preceding or during disease pathogenesis. To address this, multiple sample should be collected over time and analysed using modelling techniques to understand the longitudinal trajectory of the CRP levels. The use of longitudinal trajectory modelling methods is becoming increasingly common^26^ in health and medical research^27–30^. Trajectory modelling is especially useful when investigating associations of an outcome variable against long-term trends of one or more explanatory variables. For example, using trajectory modelling, the Avon Longitudinal Study of Parents and Children found that axial length and corneal curvature are associated with height and weight growth during early childhood^30^. In CRP-related studies, several investigations have modelled long-term CRP trajectory and found associations with limitations in activities of daily living in older adults^26^, incident diabetes^31^, and cardiovascular disease-related deaths^31^.

The primary aim of the study was to explore the choroidal and retinal thicknesses at the macular region against a 7-year trajectory of serum CRP levels in a cohort of young health adults. This will allow us to establish if a chronic, subclinical inflammatory state over time may be related to variations in choroidal or retinal thicknesses early in adulthood. In view of the reported thinner choroidal or retinal thickness with exposure to cigarette smoke in middle-aged and older smokers, by using data from childhood and early adulthood, a secondary aim was to investigate if this association might be identified earlier in the lifespan than previously recognised.

## Results

### Study sample

**Figure 1:**
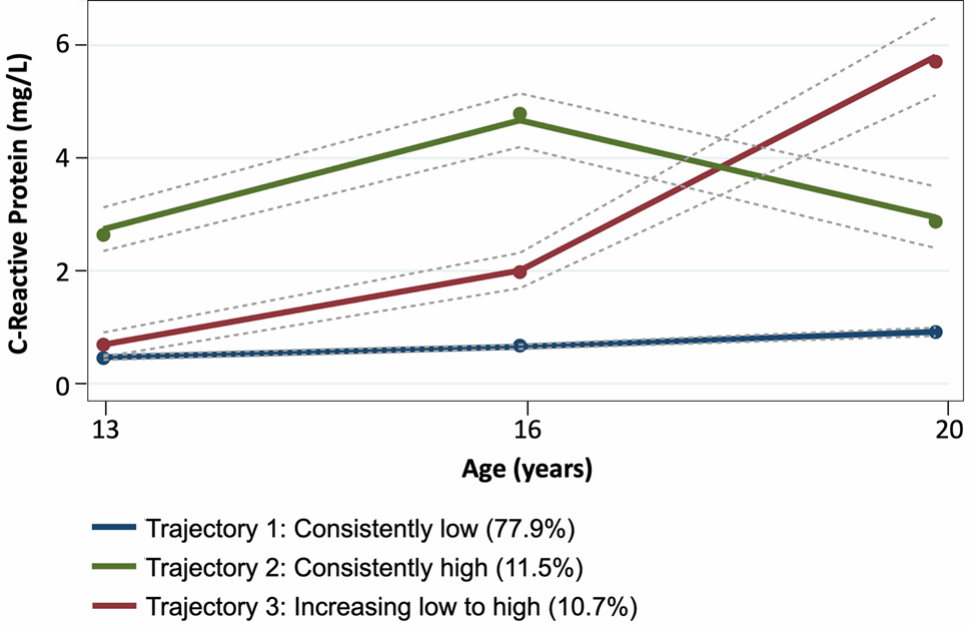
C-reactive protein trajectory groups with 95% confidence interval.

Of the original cohort of 2686 Raine Study Gen2 participants, 1260 had valid CRP data for trajectory modelling. Three trajectory groups (Fig. 1 and Supplementary Figure S1) were identified by group based trajectory modelling (GBTM). The majority of participants were in Trajectory 1 and had consistently relatively lower levels of serum CRP (labelled “Low”; 77.9%). The rest of the participants were grouped to Trajectory 2, where serum CRP levels increased between the three time-points (labelled “Low to high”; 11.5%), or Trajectory 3, where CRP levels remained consistently relatively higher (labelled “High”; 10.7%). The 95% confidence intervals for the estimated trajectory proportions and the actual trajectory membership assigned proportions are shown in Table 1.

**Table 1:** Estimated group proportions and assigned group membership proportions for the 3 CRP trajectories.

Trajectory group	1. Consistently low	2. Increasing low to high	3. Consistently high
GBTM-predicted group proportions (95%CI)	77.9% (74.9% to 80.9%)	10.7% (7.7% to 13.6%)	11.5% (8.8% to 14.1%)
Assigned group membership proportions (n, %)	1014 (80.5%)	109 (8.6%)	137 (10.9%)
Group average posterior probability	94.9%	78.9%	85.5%
**CRP levels (mg/L; median [IQR])**			
14-year	0.40 [0.23 to 0.80]	0.49 [0.30 to 0.88]	0.76 to 4.25]
17-year	0.41 [0.18 to 0.88]	1.56 [0.73 to 3.18]	5.04 [3.29 to 7.12]
20-year	0.62 [0.29 to 1.25]	5.82 [4.58 to 7.94]	2.47 [1.10 to 3.95]

CI = confidence interval; CRP = C-reactive protein; GBTM = group-based trajectory modelling; IQR = interquartile range.

**Figure 2:**
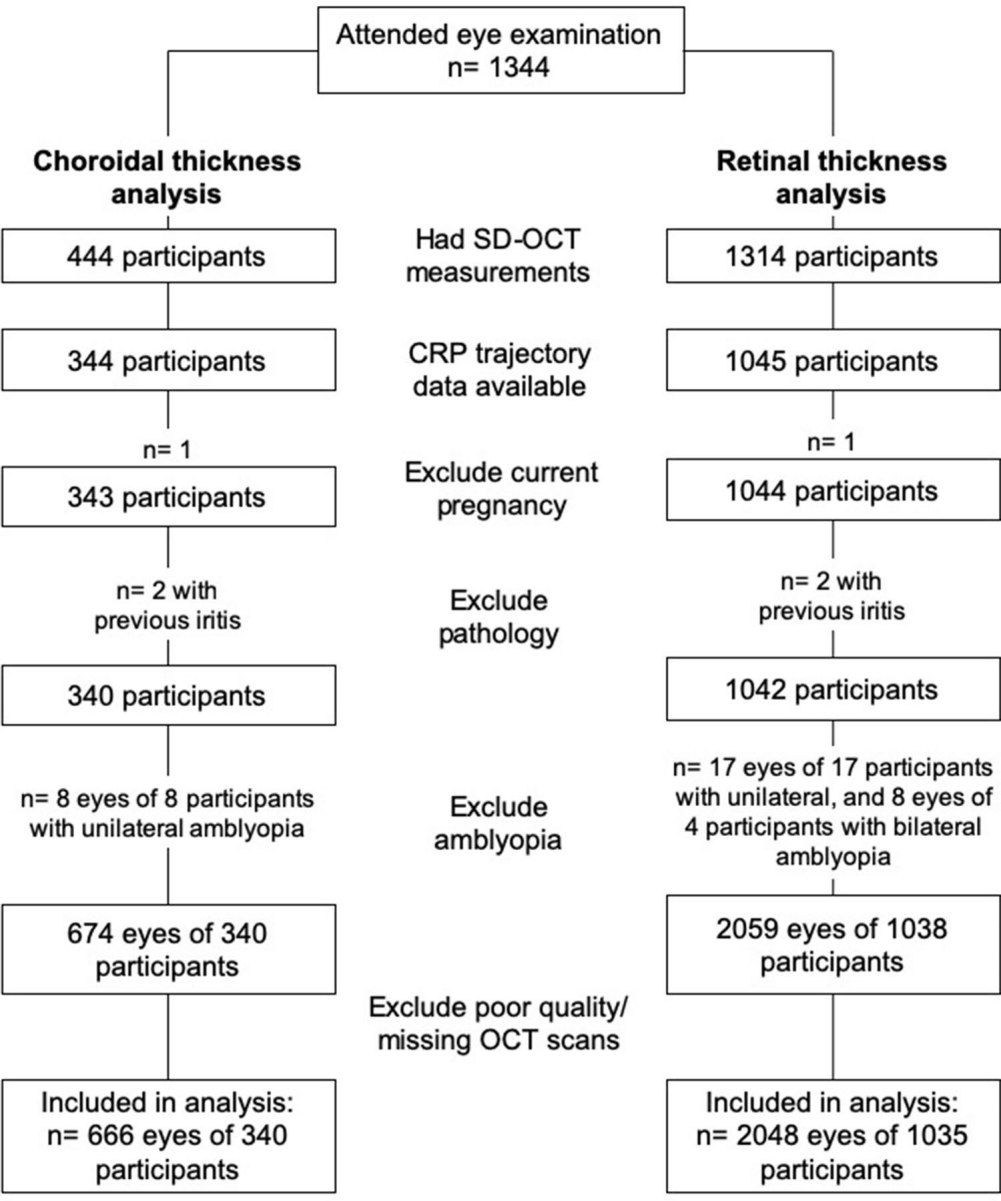
Study sample for choroidal (left) and retinal thickness (right) dataset.

After excluding those with a history of ocular pathology, eyes with amblyopia, poor quality SD-OCT scans (including truncated or decentre, scans), or current pregnancy, a total of 666 eyes of 340 participants (169 female; 49.7%) were included in the choroidal thickness analyses, while 2048 eyes of 1035 participants (492 female; 47.5%) were included in the retinal thickness analyses (Fig. 2). Participants were 18 to 22 years old at the time of the eye examination and the majority are Caucasians. Among the participants included in the choroidal thickness analysis, there were 1 (0.3%), 35 (10.2%), and 62 (18.1%) participants with diabetes, asthma, or some form of allergy (including hayfever), respectively, at the time of the eye examination. Of those included in the retinal thickness analyses, 4 (0.3%), 130 (9.9%), and 204 (15.5%) have diabetes, asthma, or some form of allergy (including hayfever) at the time of eye examination. As a purpose of this study is to investigate how chronic subclinical inflammation (which can be influenced by these systemic conditions) could be associated with eye measures, these participants were not removed from the analyses.

There was no significant difference in sex, ethnicity, pack-years smoked, exposure to secondhand smoke during childhood, body-mass index (BMI), rates of diabetes, asthma, or allergies, level of education, or annual family income between those included and excluded from the analyses (*p* ≥ 0.09; data not shown). However, those with choroidal thickness data were younger than those without, although the difference was minute (19.9 ± 0.5 vs 20.1 ± 0.4 years, *p* < 0.001). This is a result of the study participants being invited to participate in the follow-up according to date of birth; thus, younger participants attended the study visit later when the Enhance Depth Imaging (EDI) mode on the spectral-domain optical coherence tomography (SD-OCT) was available to measure choroidal thickness. Ocular measures, including axial length and intraocular pressure, similarly did not differ significantly between those included and excluded from the analyses (*p* ≥ 0.78; data not shown). Given the large difference in number of participants who had available choroidal thickness and retinal thickness measurements, the characteristics of participants included in each of these analyses are presented separately in Tables 2 and 3.

**Table 2:** Study sample characteristics for the choroidal thickness analyses (n = 341).

	CRP trajectory group		
	Low (n = 259)	Low to high (n = 36)	High (n = 45)
Age† (years; mean ± 1 SD)	19.9 ± 0.5	19.8 ± 0.4	19.8 ± 0.4
Female‡ (n, %)	121 (46.7%)	25 (69.4%)*	23 (51.1%)
**Ethnicity**§ **(n, %)**			
Caucasian	213 (82.2%)	30 (83.3%)	41 (91.1%)
East Asian	11 (4.2%)	1 (2.8%)	0 (0.0%)
South Asian	4 (1.5%)	1 (3.3%)	0 (0.0%)
Other/mixed	31 (11.9%)	4 (11.1%)	4 (8.9%)
BMI† (kg/m^2^; mean ± 1 SD)	23.9 ± 4.1	23.6 ± 5.4*	28.9 ± 7.3*
**Annual family income in AUD1,000 (n, %)§**			
< 24.0	46 (17.8%)	2 (5.6%)	10 (22.2%)
24.0 to < 36.0	59 (22.8%)	8 (22.2%)	13 (28.9%)
> 36.0	83 (32.0%)	13 (36.1%)	9 (28.9%)
No response / prefer not to say	71 (27.4%)	13 (36.1%)	13 (29.9%)
**Highest education (n, %)§**			
Primary	4 (1.5%)	0 (0.0%)	1 (0.0%)
Secondary	157 (60.6%)	25 (69.4%)	27 (60.0%)
University	14 (5.4%)	2 (5.6%)	1 (2.2%)
Other / prefer not to say	84 (32.4%)	9 (24.9%)	17 (37.8%)
Smokers§ (n, %)	25 (9.7%)	3 (8.3%)	7 (15.6%)
Pack-years smoked^||^ (median [IQR]; range)	0.0 [0.0 to 0.0] Range = 0.0 to 2.2	0.0 [0.0 to 0.0] Range = 0.0 to 0.5	0.0 [0.0 to 0.0] Range = 0.0 to 4.1
**Childhood exposure to secondhand smoke (n, %)‡**			
Limited exposure	131 (51.7%)	20 (55.6%)	16 (40.0%)
Some/heavy exposure	125 (48.3%)	16 (55.6%)	27 (60.0%)
**Ocular measures# (median [IQR])**			
Spherical equivalent (D)	+ 0.38 [− 0.25 to + 0.63]	+ 0.25 [− 0.13 to + 0.63]	+ 0.38 [− 0.09 to + 0.75]
Axial length (mm)	23.5 [23.0 to 24.0]	23.4 [23.0 to 23.8]	23.4 [23.8 to 24.0]
IOP (mmHg)	16 [14 to 19]	16 [13 to 17]	16 [14 to 19]

BMI = body mass index; CRP = c-reactive protein; IOP = intraocular pressure; IQR = interquartile range; SD = standard deviation. *Statistically different from the “Low” group at *p* < 0.001; †group difference analysed using one-way analysis of variance; ‡analysed using Chi-squared test; §analysed using Fisher exact test; ^||^range additionally shown as median and IQR values are all 0, analysed using the using the Kruskal–Wallis test; #analysed using generalised estimating equations.

**Table 3:** Study sample characteristics for retinal thickness analyses (n = 1036).

	CRP trajectory group		
	Low (n = 831)	Low to High (n = 97)	High (n = 107)
Age† (years; mean ± 1 SD)	20.1 ± 0.5	20.0 ± 0.3	20.0 ± 0.4
Female‡ (n, %)	369 (44.4%)	69 (71.1%)*	54 (50.5%)
**Ethnicity§ (n, %)**			
Caucasian	708 (85.2%)	85 (87.6%)	95 (88.8%)
East Asian	22 (2.6%)	1 (1.0%)	1 (0.0%)
South Asian	11 (1.3%)	2 (2.4%)	2 (1.1%)
Other/mixed	90 (10.8%)	9 (9.3%)	11 (10.3%)
BMI† (kg/m^2^; mean ± 1 SD)	23.9 ± 3.9	26.1 ± 5.4*	29.3 ± 7.6*
**Annual family income in AUD1,000 (n, %)§**			
3 < 24.0	261 (31.4%0	28 (28.9)	43 (40.2%)
4 24.0 to < 36.0	217 (26.1%)	21 (21.6%)	28 (26.2%)
5 > 36.0	312 (37.5%)	44 (45.5%)	30 (28.0%)
6 No response / prefer not to say	41 (4.9%)	4 (4.1%)	6 (5.6%)
**Highest education (n, %)**			
7 Primary	12 (1.4%)	1 (1.0%)	3 (2.8%)
8 Secondary	504 (60.6%)	61 (62.9%)	61 (57.0%)
9 University	45 (5.4%)	5 (5.2%)	4 (3.7%)
10 Other / prefer not to say	170 (32.2%)	30 (31.0%)	39 (36.4%)
Smokers^§^ (n, %)	97 (11.7%)	11 (11.3%)	17 (15.9%)
Pack-years smoked||(median [IQR] and range)	0.0 [0.0 to 0.0] Range = 0.0 to 2.3	0.0 [0.0 to 0.0] Range = 0.0 to 0.5	0.0 [0.0 to 0.0] Range = 0.0 to 4.1
**Childhood exposure to secondhand smoke (n, %)‡**			
10 Limited exposure	434 (52.2%)	45 (46.4%)	42(39.3%)
11 Some/heavy exposure	397 (47.8%)	52 (46.4%)	65 (60.7%)
**Ocular measures# (median [IQR])**			
Spherical equivalent (D)	+ 0.25 [− 0.38 to + 0.63]	+ 0.13 [− 0.38 to + 0.63]	+ 0.25 [− 0.25 to + 0.63]
Axial length (mm)	23.5 [23.1 to 24.1]	23.3 [23.0 to 23.9]	23.5 [23.0 to 24.0]
IOP (mmHg)	15 [13 to 18]	14 [12 to 17]*	15 [13 to 17]

BMI = body mass index; CRP = c-reactive protein; IOP = intraocular pressure; IQR = interquartile range; SD = standard deviation.

*Statistically different from the “Low” group at *p* < 0.001; †group difference analysed using one-way analysis of variance; ‡analysed using Chi-squared test; §analysed using Fisher exact test; ||range additionally shown as median and IQR values are all 0, analysed using the using the Kruskal–Wallis test; #analysed using generalised estimating equations.

For the sample included in the choroidal thickness analysis (Table 2), there were significantly more females in the “Low to high” CRP trajectory group compared to the “Low” group, while the average body mass index (BMI) was statistically different between all three groups. There was no difference in number of smokers, pack-years, ethnicity, or ocular measures between groups.

For the retinal thickness analysis (Table 3), there were similarly more females in the “Low to high” group compared to the “Low” group, and BMI was significantly different between all three trajectory groups. Moreover, a significantly higher proportion of participants in the “High” group had “Some or heavy exposure” to secondhand smoke during childhood (*p* = 0.016). The “Low to high” group had, on average, higher IOPs than the other two groups. There was no other significant difference in participant demography between groups (*p* < 0.05).

### C-reactive protein trajectory

**Figure 3:**
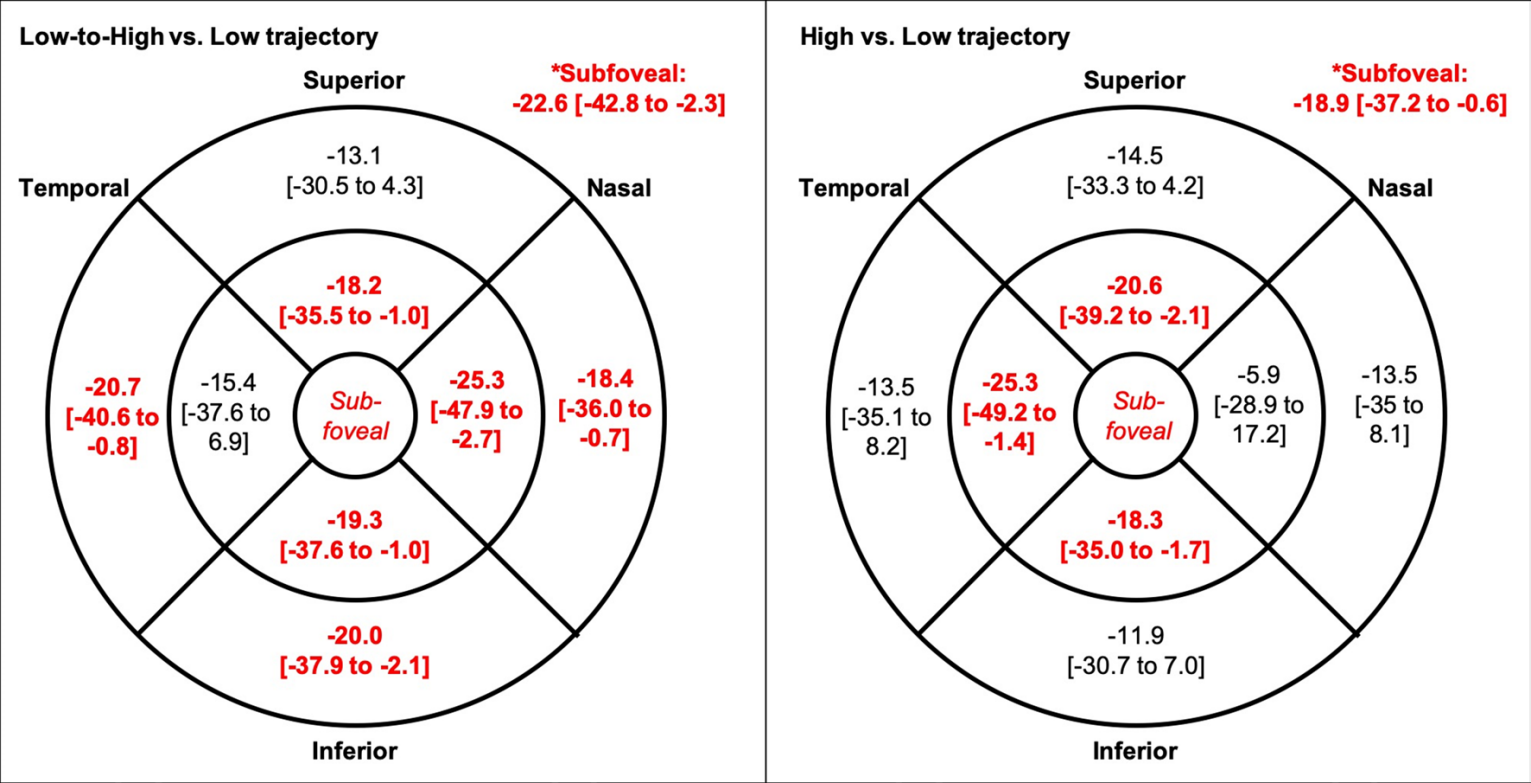
Mean difference [and 95% confidence interval] in choroidal thickness (μm) at the central (0.5-mm radius around the fovea), inner (between 0.5- and 1.5-mm radius around the fovea), and outer macula between (1.5- and 3.0-mm radius around the fovea) between c-reactive protein trajectory groups. Statistically significant positive associations shown in bold and red (*p* < 0.01), corrected for sex, ethnicity, BMI, ocular measures, exposure to cigarette smoke, and time of day of SD-OCT imaging. Reference = “Low” trajectory.

Table 4 shows the analyses for the associations between central choroidal thickness and 7-year CRP trajectory. On average, participants in the “Low to high” or “High” trajectory groups had significantly thinner choroids in most regions of the macula, including the central macula, compared to those in the “Low” group (Fig. 3). There was no significant difference in choroidal thickness at any region between the “Low to high” and “High” groups (*p* > 0.05). Retinal thickness did not differ between groups at any region (*p* > 0.05).

**Table 4:** Association between central choroidal thickness* (μm) and 7-year CRP trajectory.

	Crude analysis			Fully adjusted^*#*^		
	Estimate [95%CI]	Wald χ^2^	*p*-value	Estimate [95%CI]	Wald χ^2^	*p*-value
*CRP trajectory group (Ref* = *Low)*						
Low-to-high	− 24.4 [− 40.0 to − 8.7]	9.3	0.002§	− 22.6 [− 42.8 to − 2.3]	4.8	0.029†
High	− 10.6 [− 24.2 to 3.0]	2.3	0.13	− 18.9 [− 37.2 to − 0.36]	4.2	0.043†
Male (Ref = female)	**–**	**–**	**–**	19.4 [5.3 to 33.4]	7.3	0.007‡
*Ethnicity (Ref* = *Caucasian)*						
East Asian	**–**	**–**	**–**	− 8.9 [− 37.4 to 19.53]	0.4	0.54
South Asian	**–**	**–**	**–**	− 18.6 [− 70.9 to 33.9]	0.5	0.49
Other/mixed	**–**	**–**	**–**	− 12.8 [− 31.5 to 5.9]	1.8	0.18
BMI (kg/m^2^)	**–**	**–**	**–**	− 0.03 [− 1.45 to 1.39]	0.0	0.96
Pack-years smoked	**–**	**–**	**–**	− 6.2 [− 16.5 to 29.0]	0.3	0.59
Childhood exposure to secondhand smoke§	**–**	**–**	**–**	4.4 [− 10.1 to 19.0]	0.4	0.55
Time of day of SD-OCT imaging (hour)^~^	**–**	**–**	**–**	0.7 [− 0.1 to 1.5]	3.1	0.08
Axial length (mm)	**–**	**–**	**–**	− 21.8 [− 30.7 to − 12.9]	23.1	< 0.001‡
IOP (mmHg)	**–**	**–**	**–**	− 0.3 [− 1.2 to 1.4]	0.2	0.66

BMI = body mass index; CI = confidence interval; CRP = c-reactive protein; IOP = intraocular pressure; SD-OCT = spectral domain optical coherence tomography. *Central choroid defined as 0.5 mm radius around fovea; ^#^adjusted for all variables in table; § some or heavy exposure to secondhand smoke from 1- to 14-years old, with reference to no or limited exposure; ^~^In 24-h notation. †*p* < 0.05; ‡*p* < 0.01.

## Exposure to cigarette smoke

**Figure 4:**
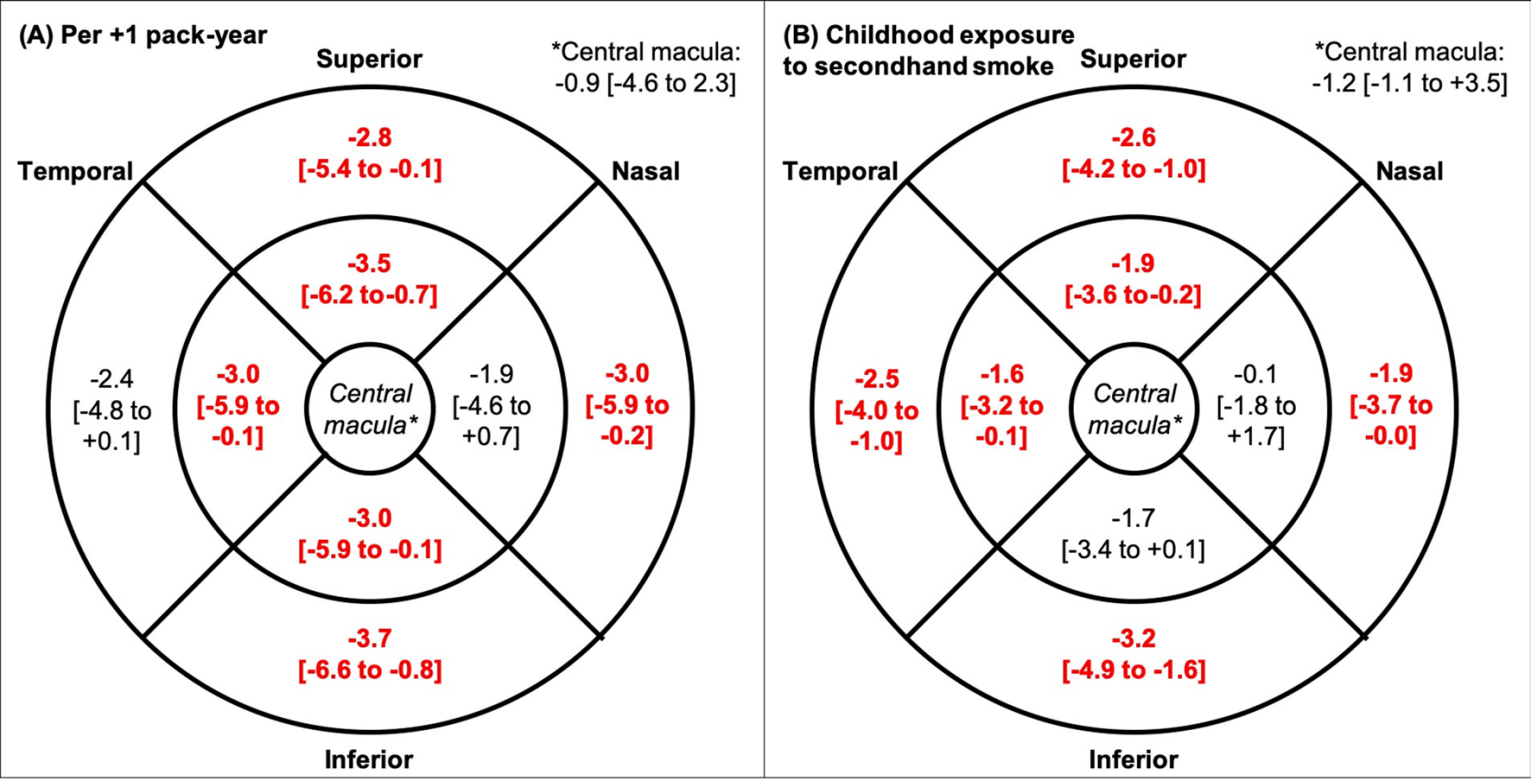
Mean difference [and 95% confidence interval] in retinal thickness (μm) and at the central (0.5-mm radius around the fovea), inner (between 0.5- and 1.5-mm radius around the fovea), and outer macula between (1.5- and 3.0-mm radius around the fovea) with every additional pack-year smoked (A; left) and some or heavy exposure to secondhand smoke during childhood (B; right). Statistically significant positive associations in bold and red (*p* < 0.05), corrected for sex, ethnicity, BMI, and ocular measures.

Pack-years smoked had no significant association with choroidal thickness at any region or with the retinal thickness at the central macula. However, the retina was thinner at most other macular regions with higher pack-years smoked (Fig. 4). This remained significant (*p*-value range = 0.014 to 0.045) after correcting for sex, ethnicity, BMI and ocular measures.

Similarly, childhood exposure to secondhand smoke was not associated with choroidal thickness, but was significantly associated with thinner retina at most non-central macular regions, after adjusting for sex, ethnicity, BMI, and ocular measures (*p* = 0.001 to 0.030; Fig. 4).

## Discussion

In this study, we demonstrated that consistently high or increasing serum CRP levels over a 7-year period was associated with thinner choroids, one of the most vascular tissues in the eye. This finding aligns with those of previous studies on individuals with systemic autoimmune diseases where longer duration of disease activity, and thus prolonged duration of elevated CRP levels, was linked to thinner subfoveal choroids^5,32^. Park et al.^5^ estimated that the subfoveal choroid is thinner by 0.44 μm for each additional month of inflammatory disease activity. Our observation suggests that chronic inflammation, as measured using CRP levels, may be linked with thinner choroidal thicknesses since young adulthood, even if the inflammation is subclinical. Although the estimated thinning is small, about 19 to 23 µm or roughly 5 to 6% of the normal subfoveal choroidal thickness in healthy young adults^33^, as the choroid continues to thin with consistently high or increasing CRP levels, its ability to supply the outer retina may be reduced over time, possibly increasing the risk of future AMD from a young age.

On the other hand, we failed to find a significant difference in retinal thickness between trajectory groups. Previous studies involving patients with a history of autoimmune conditions, including chronic uveitis^34^ or systemic inflammation^6,7^, have found thinner retinas in the patient group relative to controls. The lack of significant group difference in the current study may suggest that the presence of subclinical inflammation may not influence retinal thickness, at least in early adulthood. It is possible that elevated CRP levels may take a longer time to influence the retinal thickness, and that choroidal changes precedes retinal thinning. Even though the CRP levels were documented over a fairly long duration in the current study (7 years), our study participants are young and any significant effects of high or increasing CRP trajectories on retinal thickness may only be apparent after a longer duration.

While studies have agreed on the impact of cigarette smoking on the choroid^23,35^, our analysis did not reveal a significant association between choroidal thickness and pack-years smoked. The young age of our participants, and thus relatively low pack-years, may account for the lack of statistical association. However, a recent study^35^ found thinner choroids in 1400 children 6- to 8-years of age who had been exposed to second-hand smoke, compared to those with no exposure, suggesting that the choroid can be affected by cigarette smoking from childhood. Thus, the lack of association in our study is more likely due to the relatively small sample of participants with choroidal thickness data available for analysis.

Smoking is a well-established risk factor of AMD; Klein et al.^36^ reported that the odds of having AMD is increased by 2% for every pack-year smoked, while another study^37^ found that 40 pack-years of smoking was linked with a 3.4 and 2.5 increased odds of geographical atrophy and choroidal neovascularisation, respectively. In our study, the smokers have a median of only 0.37 pack-years, a low number attributed to the young age of our participants. Based on the findings of these previous studies, the risk of AMD in the smokers in our study sample may not be significantly elevated yet^36,37^. However, we observed a significant inverse association between pack-years and full retinal thickness at most non-central regions of the macula, such that the retina thins on average by ~ 3 μm (roughly 1% of the full thickness), depending on the macular region, with each additional pack-year smoked. Similarly, exposure to secondhand smoke during childhood was associated with thinner retinas by 2 to 3 µm, in agreement with previous observations. In a small case–control study, Wood et al.^9^ reported that those with early AMD had significantly thinner retinas by 11 to 30 μm, depending on the macular region, compared with age-matched healthy controls, suggesting that retinal thinning may be a manifestation of early AMD^9^. The significant association highlights the possibility that smoking may affect retina morphology even from a young age. If these participants continue smoking, the increasing pack-years would inherently raise their risk of developing AMD, with the increased risk possibly manifesting as further retinal thinning. We will be able to investigate this as we follow this cohort of participants through the next few decades.

The implications of our study findings have to be considered in light of its strengths and limitations. The 7-year CRP trajectory data allowed us to assess the effect of long-term exposure to elevated levels of the inflammatory marker, which is superior to using CRP measurements at a single time-point. Moreover, we had information on participant smoking habits, from which the pack-year measure could be derived. Rather than categorising participants into current/former/non-smokers or light/moderate/heavy smokers, pack-years accounts for the duration and quantity smoked. However, many participants had to be excluded from the analyses due to a lack of choroidal thickness or CRP trajectory information, which may be a main limitation of the study. Moreover, as we only had ocular measurements during the 20-year follow-up of the Raine study, we were not able to determine a causal relationship between CRP trajectory, smoking, and ocular measures. Longitudinal follow-ups of the Raine Study participants are underway to address this.

In conclusion, our findings support the hypothesis that chronic subclinical inflammation, as suggested by relatively high or increasing CRP levels over a 7-year period, may affect choroidal morphology from young adulthood. Moreover, there an association between higher pack-years smoked or childhood exposure to secondhand smoke and thinner retina even at 18–22 years of age. While the differences in choroidal or retinal thickness with CRP trajectory or smoking exposure are not clinically significant enough to conclude any disease diagnosis or affect the vision of these young adults, they highlight a possibility that an increased risk of visual impairment associated with these factors may be present since young adulthood. As we follow this cohort of young adults through the next several decades, we will be able to determine the long-term implications of elevated CRP levels on ocular health, in particular to its relation to AMD incidence and whether choroidal or retinal thinning early in adulthood precedes macular pathology.

## Methods

### Study sample

This was conducted as part of the Raine Study^38^, a multigeneration, longitudinal study that has been following an original cohort of participants (Gen2 participants of the Raine Study) since their prenatal period. Findings from the eye examination of the Raine Study have revealed associations between choroidal thickness and best-corrected visual acuity^39^ and between obstructive sleep apnoea and thinner retinal nerve fibre layers in young adult ^40^, in addition to several other findings on the link between environmental or early-life factors and ocular parameters during young adulthood^41–45^. Between 1989 and 1992, 2,686 individuals (Gen2) were born to 2,900 women (Gen1) at the King Edward Memorial Hospital in Perth, Western Australia. These offspring have since been undergoing a series of health and medical tests and completed questionnaires at the 1-, 2-, 3-, 5-, 8-, 10-, 13-, 16-, and 20-year follow-ups of the study. Blood samples were collected from participants at the Gen2 13-, 16-, and 20-year follow-ups, from which high-sensitivity CRP levels were obtained. Participants additionally underwent a comprehensive eye examination at the Gen2 20-year follow-up.

Participants with a self-reported diagnosis of posterior segment disease or who were found to have a current retinal or optic disc pathology during the eye examination were excluded from the analysis. Given that retinal and choroidal thicknesses have been reported to be thinner in autoimmune disease^6^, individuals with a history of any uveitis were also excluded. Amblyopic eyes, defined as best-corrected visual acuity of < 6/9 (~ 0.18 logarithm of minimum angle of resolution [logMAR])^46^ were also removed from the analysis as studies have reported thicker retinas^47^ and choroids^48^ in amblyopic eyes.

Prior to each follow-up visit, participants were given a full explanation of the nature of the study and provided written informed consent. All follow-ups of the Raine study had been approved by the University of Western Australia’s Human Research Ethics Committee, and were conducted in accordance with the tenets of the Declaration of Helsinki.

### High sensitivity CRP analysis

At the Gen2 13-, 16-, and 20-year follow-ups, overnight-fasted blood samples were collected in the morning by a single experienced phlebotomist. Samples were then transferred to be stored at − 80 °C within 2 h of collection. The serum was extracted from the samples and analysed in daily batches of 100 using an immunoturbidimetric method on an Architect c16000 Analyser (Abbott core Laboratory, Illinois, United States of America)^49^. The immunoassays for high-sensitivity CRP are known to be well standardised, robust, reproducible, and have low inter-examiner effects^11^, and the immunoturbidimetric method has been found to correlate well with representative immunopheloetric assays^50^. The maximum time between sample collection and analysis was one month, which does not affect CRP levels.

### CRP trajectory modelling

STATA 16.1 (StataCorp, Texas, USA; https://www.stata.com) was used to create group-based trajectories of CRP data for the three time-points (13-, 16-, and 20-year follow-ups). The STATA ‘TRAJ’ plug-in module was utilised for this purpose. GBTM is a person-centred approach to identify groups of individuals with certain attributes (as opposed to a variable-centred approach that aims to describe associations between variables). It is a form of finite mixture modelling using maximum likelihood to create trajectories of average values within homogenous subgroups of individuals^51^.

Data points of CRP values > 10 mg/L were removed from the trajectory modelling to rule out acute inflammation and/or current infection^26^. Included participants had at least two valid measures of CRP over the three follow-ups. Models were systematically created to investigate the number of trajectory groups and the polynomial shape of the trajectories. Model rendering began with the simplest solution (two groups) and increased until the Bayesian Information Criterion plateaued and every group comprised ≥ 5% of the participants^51^. There are five well established a priori diagnostic criteria for best fit in GBTM, including: (1) mean posterior probability ≥ 70% for each trajectory, (2) odds of correct classification ≥ 5 for each trajectory, (3) close approximation between the estimated trajectory proportions and the assigned membership proportions, (4) reasonably tight confidence intervals around estimated values, and (5) meaningful distinction between the trajectories^51^. Trajectory modelling was performed independent of the eye measures.

### Exposure to cigarette smoking

During the participants’ childhood, at the Gen2 1-, 2-, 3-, 5-, 8-, 10-, and 14-year follow-ups, the primary carer of the participants completed a questionnaire that collected prospective information on the number of smokers in the household and the quantity smoked. Based on these responses, participants’ childhood exposure to secondhand smoke were categorised as “Limited exposure” if none of their household members smoked during the 1- to 14-year follow-ups, or “Some or heavy exposure” if they have lived with one or more smokers at any point during their childhood.

At the Gen2 20-year follow-up, when participants were 18–22 years of age, they completed a questionnaire that collected information on their smoking status, including age at which they started smoking, cigarettes smoked per day, and previous smoking. We calculated the pack-years smoked for each participant as the number of packs (of 20 cigarettes) smoked per day multiplied by the number of years they had (or had previously) been smoking.

### Eye examination

As part of the eye examination at the Gen2 20-year follow-up^52^, participants underwent post-cycloplegic autorefraction (Nidek ARK-510A Auto Refractometer, Nidek Co Ltd, Japan), ocular biometry (IOLMaster version 5; Carl Zeiss Meditec AG, Jena, Germany), intraocular pressure (IOP) measurement (ICare TA01i rebound tonometer; Icare Finland Oy, Vantaa, Finland), and spectral domain optical coherence tomography (SD-OCT) imaging (Spectralis HRA + OCT; Heidelberg Engineering, Heidelberg, Germany). Eye examinations were conducted between 10am and 7pm in the day.

**Figure 5:**
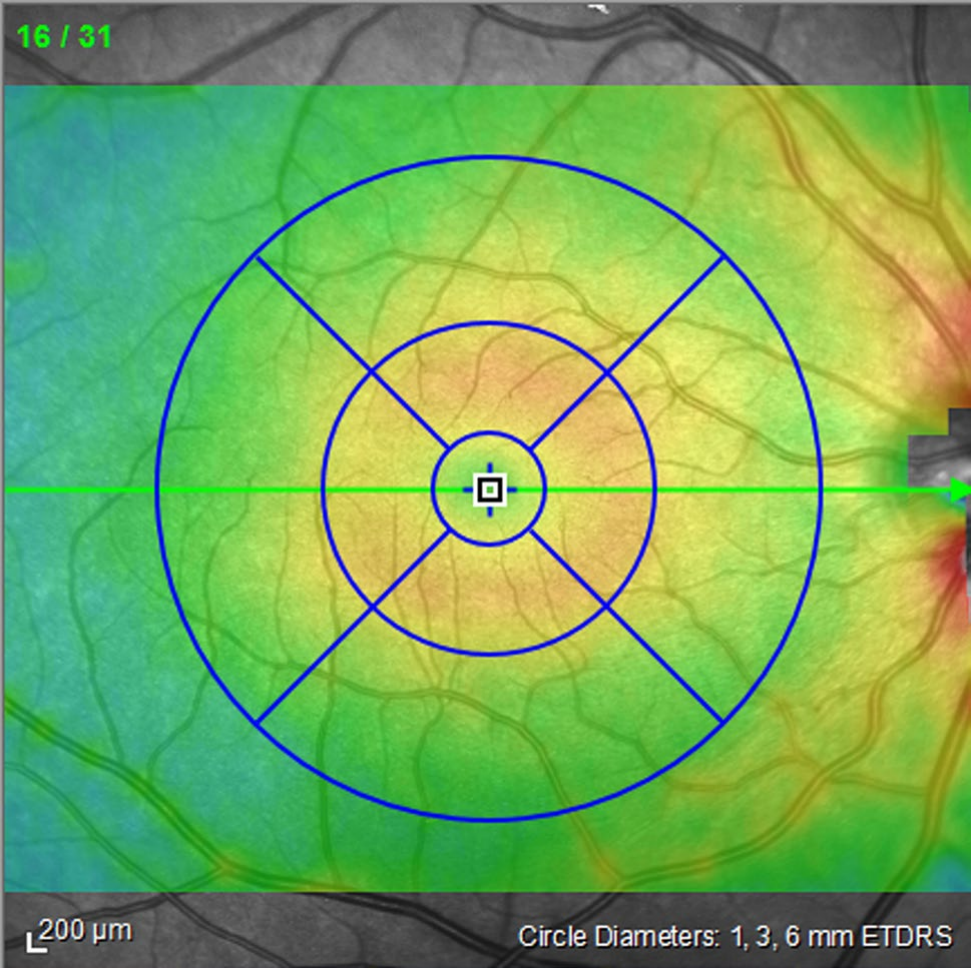
Macular regions measured: central (0.5-mm radius around the fovea), inner ring (region between 0.5- and 1.5-mm radius around the fovea), and outer ring (region between 0.5- and 3.0-mm radius around the fovea) at the superior, inferior, temporal, and nasal quadrants.

To measure the central retinal thickness, 31-raster B-scans covering a 30º × 25º area centred on the fovea were obtained from each eye, with each B-scan averaged from 9 frames. Scan quality was maintained at a signal-to-noise ratio of 20 or higher and additionally assessed subjectively by the technician when the imaging was completed. The full retinal thickness was measured at 9 macular regions according to the Early Treatment of Diabetic Retinopathy (ETDRS) grid^53^, which includes the central (0.5 mm radius around the fovea), inner ring (region between 0.5 and 1.5 mm radius around the fovea), and outer ring (region between 1.5 and 3.0 mm radius around the fovea), and at the superior, inferior, temporal, and nasal quadrants (Fig. 5). Retinal thickness measurements obtained using the Spectralis SD-OCT algorithm is known to have excellent reproducibility and repeatability with minimal inter- and intra-observer variations^54^.

To measure choroidal thickness, additional scans were taken using the enhanced depth imaging mode on the SD-OCT, which allowed us to obtain high-resolution cross-sectional images of the choroid. The detailed methodology of choroidal imaging and thickness measurement in this group of participants has been described previously^39^. In brief, 2 horizontal and 2 vertical B-scans centred on the fovea were taken. The average of 100 frames were used for each B-scan, which spanned about 30º. Images were exported and the choroidal thickness were measured offline using an automated non-commercial software that uses deep learning methods to automatically segment outer boundary of the retinal pigmented epithelium and the chorioscleral interface^55^. Choroidal thickness obtained from this automated segmentation have been shown to high agreement with manual measurements^55^. Moreover, an experienced observer, blind to the CRP trajectory and smoking status of participants, manually checked and corrected the segmentation if necessary^39^. The choroidal thickness was determined at the central, inner ring, and outer ring of the macula, as per the ETDRS grid (Fig. 5) As the enhanced depth imaging mode was only available on the SD-OCT midway through the data collection phase of the Gen2 20-year follow-up of the Raine Study, only a subset of participants had choroidal thickness data available for analysis.

### Statistical analysis

All analyses were conducted in the R Statistical Environment (version 3.6.3; 2019 The R Foundation for Statistical Computing Platform, Vienna, Austria; https://www.r-project.org/), and level of significance was set at *p* < 0.05. Continuous variables were expressed in terms of the mean and standard deviation or median and interquartile ranges, depending on their distribution. Difference in participant demography between trajectory groups were analysed using Chi-square test or Fisher exact test for categorical variables and one-way analysis of variance or Kruskal–Wallis test for continuous variables, as appropriate. The main outcomes measures were the choroidal and retinal thickness at the central macula. For all analyses that involve eye data as the outcome measure, including the analyses for the main aim and the sub-aim, the Generalized Estimating Equations method was used as it was able to account for missing data and the non-normal data. The exchangeable correlation structure implemented within the models accounted for the within-subject correlation between the two eyes^56,57^. Unadjusted models and adjusted models were generated for each analysis, with the latter accounting for known confounding variables of choroidal or retinal thickness. These include sex, ethnicity, and body-mass index (BMI)^33,58^. For the main aim to explore associations between CRP trajectory and retinal or choroidal thickness, pack-years smoked and childhood exposure to secondhand smoke were additionally included in the adjusted models. For the secondary aim to explore the effects of cigarette smoke exposure on the choroidal or retinal thickness, childhood exposure to secondhand smoke and participants’ pack-years smoked were included in separate models due to the strong association between these two variables (*p* < 0.001, independent sample t-test). Additionally, for all analyses involving choroidal thickness data as the outcome variable, time of the day at which SD-OCT imaging was conducted was included as a co-variable in the models to account for known diurnal variations in choroidal thickness^59^.

## Data availability statement

The datasets generated during and/or analysed during the current study are not publicly available due to the Raine Study’s policy. However, the codes used for statistical analyses and trajectory modelling, and the image analysis software are available from the authors upon request.

## Supplementary Information

Supplementary Information
