# Supplementary material for: Associations between seven-year C-reactive protein trajectory or pack-years smoked with choroidal or retinal thicknesses in young adults
Source: Sci Rep. 2021 Mar 17;11:6147. doi: 10.1038/s41598-021-85626-3 (PMC7969753; doi:10.1038/s41598-021-85626-3)

Associations between seven-year C-reactive protein trajectory or pack-years smoked with choroidal or retinal thicknesses in young adults

Authors: Samantha Sze-Yee Lee[1]; Darren John Beales[2]; Fred K Chen[1, 3]; Seyhan Yazar[1, 4]; David Alonso-Caneiro[5]; David A Mackey[1, 6, 7]

[1]Centre for Ophthalmology and Visual Science (incorporating Lions Eye Institute,), University of Western Australia, Western Australia, Australia

[2]School of Physiotherapy and Exercise Science, Curtin University, Western Australia, Australia

[3]Department of Ophthalmology, Royal Perth Hospital, Western Australia, Australia

[4]Single Cell and Computational Genomics Lab, Garvan Institute of Medical Research, New South Wales, Australia

[5]Queensland University of Technology (QUT), Contact Lens and Visual Optics Laboratory, Centre for Vision and Eye Research, School of Optometry and Vision Science, Queensland, Australia

[6]Centre for Eye Research Australia, University of Melbourne, Royal Victorian Eye and Ear Hospital, Victoria, Australia

[7]School of Medicine, Menzies Research Institute Tasmania, University of Tasmania, Tasmania, Australia

Supplementary figure S1. Box plots of C-reactive protein at each follow-up according to trajectory class

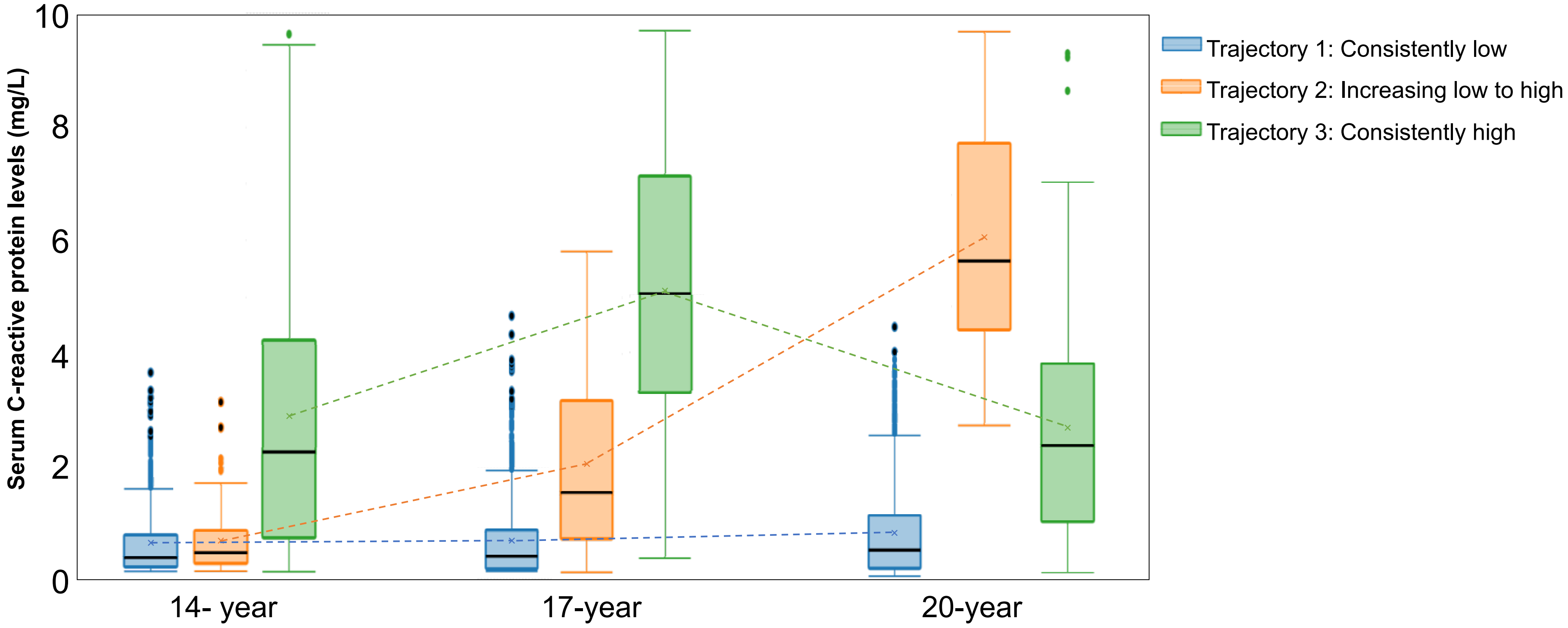

Supplement: Supplementary file 1 — Supplementary Information [file 41598_2021_85626_MOESM1_ESM.pdf]
